# Complete genome sequence of *Thermomonospora curvata* type strain (B9^T^)

**DOI:** 10.4056/sigs.1453580

**Published:** 2011-02-20

**Authors:** Olga Chertkov, Johannes Sikorski, Matt Nolan, Alla Lapidus, Susan Lucas, Tijana Glavina Del Rio, Hope Tice, Jan-Fang Cheng, Lynne Goodwin, Sam Pitluck, Konstantinos Liolios, Natalia Ivanova, Konstantinos Mavromatis, Natalia Mikhailova, Galina Ovchinnikova, Amrita Pati, Amy Chen, Krishna Palaniappan, Olivier D. Ngatchou Djao, Miriam Land, Loren Hauser, Yun-Juan Chang, Cynthia D. Jeffries, Thomas Brettin, Cliff Han, John C. Detter, Manfred Rohde, Markus Göker, Tanja Woyke, James Bristow, Jonathan A. Eisen, Victor Markowitz, Philip Hugenholtz, Hans-Peter Klenk, Nikos C. Kyrpides

**Affiliations:** 1 DOE Joint Genome Institute, Walnut Creek, California, USA; 2 Los Alamos National Laboratory, Bioscience Division, Los Alamos, New Mexico, USA; 3 DSMZ - German Collection of Microorganisms and Cell Cultures GmbH, Braunschweig, Germany; 4 Biological Data Management and Technology Center, Lawrence Berkeley National Laboratory, Berkeley, California, USA; 5 HZI – Helmholtz Centre for Infection Research, Braunschweig, Germany; 6 Oak Ridge National Laboratory, Oak Ridge, Tennessee, USA; 7 University of California Davis Genome Center, Davis, California, USA; 8 Australian Centre for Ecogenomics, School of Chemistry and Molecular Biosciences, The University of Queensland, Brisbane, Australia

**Keywords:** chemoorganotroph, facultative aerobe, eurythermal thermophile, mycelium, Gram-positive, cellulose degradation, *Thermomonosporaceae*, GEBA

## Abstract

*Thermomonospora curvata* Henssen 1957 is the type species of the genus *Thermomonospora*. This genus is of interest because members of this clade are sources of new antibiotics, enzymes, and products with pharmacological activity. In addition, members of this genus participate in the active degradation of cellulose. This is the first complete genome sequence of a member of the family *Thermomonosporaceae*. Here we describe the features of this organism, together with the complete genome sequence and annotation. The 5,639,016 bp long genome with its 4,985 protein-coding and 76 RNA genes is a part of the *** G****enomic* *** E****ncyclopedia of* *** B****acteria and* *** A****rchaea * project.

## Introduction

Strain B9^T^ (= DSM 43183 = ATCC 19995 = JCM 3096) is the type strain of *Thermomonospora curvata*, which in turn is the type species of the genus *Thermomonospora* [1]. *T. curvata* was effectively published in 1957 [1]. When the original strains R30 and R71 were no longer cultivable, strain B9 was proposed as the neotype in 1967 [2]. Currently, there are two species in the genus *Thermomonospora*, which in turn is one of the six genera in the family *Thermomonosporaceae* [3]. The generic name *Thermomonospora* was proposed by Henssen [1] for thermophilic actinomycetes isolated from composted stable manure [4]. Strain B9^T^ was isolated from municipal refuse compost samples [1]. Other (rubber degrading) strains of *T. curvata* have been isolated from food residues used in animal husbandry in Egypt (strain E4), from compost in Germany (strain E5) [5], and also from municipal solid waste compost (probably USA) [6-9]. Cellulase biosynthesis has been studied in a catabolite repression-resistant mutant of *T. curvata* [10]. Here we present a summary classification and a set of features for *T. curvata* strain B9^T^, together with the description of the complete genomic sequencing and annotation.

## Classification and features

The 16S rRNA gene sequence of the strain B9^T^ (AF002262) shows 98.1% identity with the 16S rRNA gene sequence of *T. curvata* strain E5 (AY525766) [5].The distance of strain B9^T^ to other members of this family ranged between 5% and 7%. Further analysis shows 94% 16S rRNA gene sequence identity with an uncultured bacterium, clone BG079 (HM362496) and 92% similarity to compost metagenome contig00434 (ADGO01000428) [11] from metagenomic libraries (env_nt) (status October 2010). A representative genomic 16S rRNA sequence of *T. curvata* was compared using NCBI BLAST under default settings (e.g., considering only the high-scoring segment pairs (HSPs) from the best 250 hits) with the most recent release of the Greengenes database [12] and the relative frequencies, weighted by BLAST scores, of taxa and keywords (reduced to their stem [13]) were determined. The five most frequent genera were *Actinomadura* (54.3%), *Nocardiopsis* (12.5%), *Actinocorallia* (8.8%), *Jiangella* (5.8%) and *Actinoallomurus* (5.0%) (208 hits in total). Regarding the two hits to sequences from members of the species, the average identity within HSPs was 99.9%, whereas the average coverage by HSPs was 96.2%. Regarding the single hit to sequences from other members of the genus, the average identity within HSPs was 95.2%, whereas the average coverage by HSPs was 58.4%. Among all other species, the one yielding the highest score was *Actinomadura cremea*, which corresponded to an identity of 96.3% and a HSP coverage of 85.3%. The highest-scoring environmental sequence was HM362496 ('microbial naturally composting sugarcane piles decomposting bagasse clone BG079'), which showed an identity of 94.5% and a HSP coverage of 96.3%. Within the labels of environmental samples which yielded hits, the five most frequent keywords were 'soil' (4.7%), 'compost' (3.1%), 'microbi' (2.4%), 'skin' (2.0%) and 'acid' (2.0%) (41 hits in total). These keywords partially fit to the ecology of compost and food residues, from which the known strains have been isolated [1,5,6]. Environmental samples which yielded hits of a higher score than the highest scoring species were not found.

**Figure 1:**
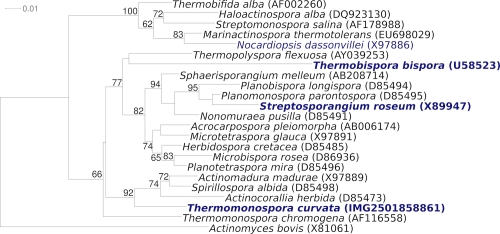
Phylogenetic tree highlighting the position of *T. curvata* relative to the type strains of the other species within the genus and to the type strains of the other genera within the suborder *Streptosporangineae*. The trees were inferred from 1,373 aligned characters [14,15] of the 16S rRNA gene sequence under the maximum likelihood criterion [16] and rooted with the type strain of the order in accordance with the current taxonomy [17]. The branches are scaled in terms of the expected number of substitutions per site. Numbers above branches are support values from 1,000 bootstrap replicates [18] if larger than 60%. Lineages with type strain genome sequencing projects registered in GOLD [19] are shown in blue, published genomes in bold [20,21].

Figure 1 shows the phylogenetic neighborhood of *T. curvata* B9^T^ in a 16S rRNA based tree. The sequences of the four 16S rRNA gene copies in the genome differ from each other by up to one nucleotide, and differ by up to five nucleotides from the previously published 16S rRNA sequence (D86945), which contains one ambiguous base call. 

**Figure 2:**
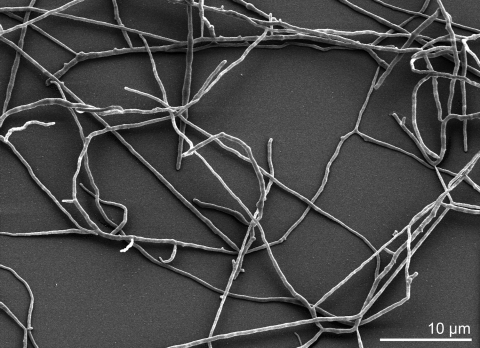
Scanning electron micrograph of *T. curvata* B9^T^

Strain B9^T^ is facultatively aerobic, Gram-positive, non-acid-alcohol-fast, and chemoorganotrophic [1,4 Table 1]. Based on the original literature, the morphology of neotype B9^T^ was the same as of the original strains [1,2]. Substrate mycelium was branched and bared aerial hyphae that differentiated into single or short chains of arthrospores [2,4] (Figure 2, arthrospores not visible). Spores were formed by the differentiation of the sporophores when they reached a given width [2]. Polymorphic and single spores in clusters appeared with a folded surface on branched and unbranched sporophores [2]. They had spindle, lemon or pear forms varying between 0.6-1.5 x 0.3-0.9 µm [2]. The optimal growth occurred at 50°C. However, weak growth was observed at 40°C and 65°C, but no growth at 28°C [2]. Colonies were white or yellow depending on culture medium [2]. On meat extract agar, the growth was moderate, aerial white mycelium formed and the colonies were yellow to brown [2]. On asparagine glucose agar, the growth was low and the aerial mycelium white [2]. On casein glucose agar, a few single colonies were observed [2]. The growth was good and the aerial mycelium white on cellulose agar medium [2]. On Czapek agar, a few spotty colonies were observed [2]. On Czapek peptone agar, the growth was good, almost no aerial mycelium formation [2]. When oatmeal agar was used as medium, the growth was good and the aerial mycelium white [2]. The growth on yeast agar was good, with thick aerial mycelium. In this case, colonies were partially yellow [2]. On yeast glucose agar, the growth was good, aerial mycelium developed later and was white while brownish colonies were formed [2]. On yeast starch agar, the growth was good, white aerial mycelium was formed and colonies were yellow to orange [2]. On potato agar I, spotty growth is observed, while no aerial mycelium was formed [2]. Few single colonies formed on potato agar II [2]. On starch agar medium, the growth was moderate and aerial mycelium was white [2]. Strain B9^T^ showed endogluconase activity and attacks cellulose [4]. It was also active in the decomposition of municipal waste compost [6-9]. When grown on protein-extracted lucerne fiber compound, strain B9^T^ released 16 times more β-glucosidases compared to growth on cellulose or purified cellulose [34]. Strain B9^T^ grew well at pH 7.5 on any nutrient medium that contains some yeast extract. It showed significant growth even at pH 11 [35]. Tests of the nitrate reduction and phosphatase were positive [4]. The sole carbon sources (1%, w/v) were ribose and sucrose. L-arabinose, galactose, lactose and mannitol were not used [4]. Strain B9^T^ was able to degrade agar, cellulose powder (MN300), carboxymethylcellulose, keratin, xylan, starch, Tween 20 and Tween 80 [4]. Growth was also observed in the presence of crystal violet (0.2 µg/ml), but it was inhibited by kanamycin and novobiocin (each 25µg/ml) [4]. The inability to utilize pectin is an important feature that differentiates strain B9^T^ from other members of the genus *Thermomonospora*. Amylases of the strain B9^T^ were extremely active and stable at 60-70°C and slightly acid to neutral pH [36-38]. Also, endoglucanase and exoglucanase were active in the strain [39]. Cellobiose was found to be a good cellulase inducer [40].

**Table 1:** Classification and general features of *T. curvata* B9^T^ according to the MIGS recommendations [22]

**MIGS ID**	**Property**	**Term**	**Evidence code**
	Current classification	Domain *Bacteria*	TAS [23]
		Phylum *Actinobacteria*	TAS [24]
		Class *Actinobacteria*	TAS [25]
		Order *Actinomycetales*	TAS [25-28]
		Family *Thermomonosporaceae*	TAS [25,28,29]
		Genus *Thermomonospora*	TAS [1,27,30,31]
		Species *Thermomonospora curvata*	TAS [1,27]
		Type strain B9	TAS [2]
	Gram stain	positive	TAS [1]
	Cell shape	mycelium	TAS [1]
	Motility	not mobile	NAS
	Sporulation	yes	TAS [1]
	Temperature range	40°C-65°C	TAS [1]
	Optimum temperature	50°C	TAS [1]
	Salinity	not reported	NAS
MIGS-22	Oxygen requirement	facultative aerobic	TAS
	Carbon source	ribose and sucrose	TAS [4]
	Energy source	chemoorganotroph	TAS [1]
MIGS-6	Habitat	compost, overheated vegetable material, straw	TAS [4]
MIGS-15	Biotic relationship	not reported	NAS
MIGS-14	Pathogenicity	no	TAS [4]
	Biosafety level	1	TAS [32]
	Isolation	rye straw	TAS [1]
MIGS-4	Geographic location	unknown, but most probably Berlin, Germany	TAS [1]
MIGS-5	Sample collection time	1959	TAS [1]
MIGS-4.1 MIGS-4.2	Latitude Longitude	52.5 13.4	NAS
MIGS-4.3	Depth	not reported	NAS
MIGS-4.4	Altitude	approx. 34-115 m above sea level	NAS

Evidence codes - IDA: Inferred from Direct Assay (first time in publication); TAS: Traceable Author Statement (i.e., a direct report exists in the literature); NAS: Non-traceable Author Statement (i.e., not directly observed for the living, isolated sample, but based on a generally accepted property for the species, or anecdotal evidence). These evidence codes are from of the Gene Ontology project [33]. If the evidence code is IDA, then the property was directly observed by one of the authors or an expert mentioned in the acknowledgements

## Chemotaxonomy

Strain B9^T^ possesses a cell wall type III with A1γ and with *meso*-diaminopimelic acid as major constituent [4,41,42]. The principal menaquinones are MK-9(H_4_), MK-9(H_6_) and MK-9(H_8_), with MK-9(H_6_) being the predominant one (the profile type *sensu* Kroppenstedt is termed 4B2 [43]) [4]. The fatty acid profile was described to be of type 3a [4]. Members of this type can synthesize terminally branched and 10-methyl-branched fatty acids [43]. *T. curvata* lacks madurose, a type C sugar and has polar lipids of type IV [4], represented by phosphatidylinositol (PI) and unknown phospholipids (PL), which according to Lechevalier [44,45] are, phosphatidyglycerol (PE), phosphatidylinositolmannosides(PIM) and diphosphatidylglycerol (DPG) [4].

## Genome sequencing and annotation

### Genome project history

This organism was selected for sequencing on the basis of its phylogenetic position [46], and is part of the *** G****enomic* *** E****ncyclopedia of* *** B****acteria and* *** A****rchaea * project [47]. The genome project is deposited in the Genomes OnLine Database [19] and the complete genome sequence is deposited in GenBank. Sequencing, finishing and annotation were performed by the DOE Joint Genome Institute (JGI). A summary of the project information is shown in Table 2.

**Table 2:** Genome sequencing project information

**MIGS ID**	**Property**	**Term**
MIGS-31	Finishing quality	Finished
MIGS-28	Libraries used	One Sanger 6 kb pMCL200 library, one 454 pyrosequence standard library and one Illumina standard library
MIGS-29	Sequencing platforms	ABI3730, 454 GS FLX, Illumina GAii
MIGS-31.2	Sequencing coverage	9.7 × Sanger; 26.6 × pyrosequence
MIGS-30	Assemblers	Newbler version 1.1.02.15, phrap
MIGS-32	Gene calling method	Prodigal 1.4, GenePRIMP
	INSDC ID	CP001738
	Genbank Date of Release	November 19, 2009
	GOLD ID	Gc01146
	NCBI project ID	20825
	Database: IMG-GEBA	646311963
MIGS-13	Source material identifier	DSM 43183
	Project relevance	Tree of Life, GEBA

### Growth conditions and DNA isolation

*T. curvata* B9^T^, DSM 43183, was grown in DSMZ medium 550 (CYC medium, modified following Cross and Attwell, 1973) [48] at 45°C. DNA was isolated from 0.5-1 g of cell paste using MasterPure Gram-positive NDA purification kit (Epicentre MGP04100) following the standard protocol as recommended by the manufacturer, with modification st/LALM for cell lysis as described in Wu *et al*. [47]. DNA is available through the DNA bank Network [49,50].

### Genome sequencing and assembly

The genome of was sequenced using a combination of Sanger and 454 sequencing platforms. All general aspects of library construction and sequencing can be found at the JGI website [51]. Pyrosequencing reads were assembled using the Newbler assembler version 1.1.02.15 (Roche). Large Newbler contigs were broken into 6,203 overlapping fragments of 1,000 bp and entered into assembly as pseudo-reads. The sequences were assigned quality scores based on Newbler consensus q-scores with modifications to account for overlap redundancy and adjust inflated q-scores. A hybrid 454/Sanger assembly was made using the parallel phrap (High Performance Software, LLC). Possible mis-assemblies were corrected with Dupfinisher [52] or transposon bombing of bridging clones (Epicentre Biotechnologies, Madison, WI). A total of 2,673 Sanger finishing reads were produced to close gaps, to resolve repetitive regions, and to raise the quality of the finished sequence. Illumina reads that were used to correct potential base errors and increase consensus quality using a software Polisher developed at JGI [53]. The error rate of the completed genome sequence is less than 1 in 100,000. Together, the combination of the Sanger and 454 sequencing platforms provided 36.3 × coverage of the genome. The final assembly contains 73,067 Sanger reads and 602,893 pyrosequencing reads.

### Genome annotation

Genes were identified using Prodigal [54] as part of the Oak Ridge National Laboratory genome annotation pipeline, followed by a round of manual curation using the JGI GenePRIMP pipeline [55]. The predicted CDSs were translated and used to search the National Center for Biotechnology Information (NCBI) nonredundant database, UniProt, TIGRFam, Pfam, PRIAM, KEGG, COG, and InterPro databases. Additional gene prediction analysis and functional annotation was performed within the Integrated Microbial Genomes - Expert Review (IMG-ER) platform [56].

## Genome properties

**Figure 3:**
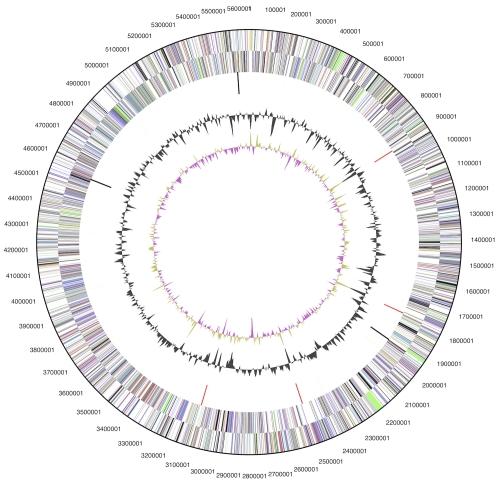
Graphical circular map of the genome. From outside to the center: Genes on forward strand (color by COG categories), Genes on reverse strand (color by COG categories), RNA genes (tRNAs green, rRNAs red, other RNAs black), GC content, GC skew.

The genome consists of a 5,639,016 bp long chromosome with a 71.6% GC content (Table 3 and Figure 3). Of the 5,061 genes predicted, 4,985 were protein-coding genes, and 76 RNAs; ninety five pseudogenes were also identified. The majority of the protein-coding genes (64.7%) were assigned with a putative function while the remaining ones were annotated as hypothetical proteins. The distribution of genes into COGs functional categories is presented in Table 4.

**Table 3:** Genome Statistics

**Attribute**	**Value**	**% of Total**
Genome size (bp)	5,639,016	100.00%
DNA coding region (bp)	4,739,306	84.04%
DNA G+C content (bp)	4,039,905	71.64%
Number of replicons	1	
Extrachromosomal elements	0	
Total genes	5,061	100.00%
RNA genes	76	1.50%
rRNA operons	4	
Protein-coding genes	4,985	98.50%
Pseudo genes	95	1.88%
Genes with function prediction	3,275	64.71%
Genes in paralog clusters	895	17.68%
Genes assigned to COGs	3,274	64.69%
Genes assigned Pfam domains	3,647	72.06%
Genes with signal peptides	1,418	28.02%
Genes with transmembrane helices	1,089	21.52%
CRISPR repeats	12	

**Table 4:** Number of genes associated with the general COG functional categories

**Code**	**value**	**%age**	**Description**
J	169	4.5	Translation, ribosomal structure and biogenesis
A	2	0.1	RNA processing and modification
K	382	10.1	Transcription
L	174	4.6	Replication, recombination and repair
B	1	0.0	Chromatin structure and dynamics
D	41	1.1	Cell cycle control, cell division, chromosome partitioning
Y	0	0.0	Nuclear structure
V	68	1.8	Defense mechanisms
T	270	7.2	Signal transduction mechanisms
M	159	4.2	Cell wall/membrane/envelope biogenesis
N	2	0.1	Cell motility
Z	2	0.1	Cytoskeleton
W	0	0.0	Extracellular structures
U	38	1.0	Intracellular trafficking and secretion, and vesicular transport
O	134	3.6	Posttranslational modification, protein turnover, chaperones
C	256	6.8	Energy production and conversion
G	193	5.1	Carbohydrate transport and metabolism
E	292	7.7	Amino acid transport and metabolism
F	78	2.1	Nucleotide transport and metabolism
H	161	4.3	Coenzyme transport and metabolism
I	265	6.8	Lipid transport and metabolism
P	160	4.2	Inorganic ion transport and metabolism
Q	181	4.8	Secondary metabolites biosynthesis, transport and catabolism
R	511	13.5	General function prediction only
S	243	6.4	Function unknown
-	1,787	35.3	Not in COGs
